# Book review of The Physics of Radiation Dosimetry by Jerome A. Meli, AIP Publishing, 2020.

**DOI:** 10.1002/acm2.13499

**Published:** 2022-01-19

**Authors:** Mats Isaksson

**Affiliations:** ^1^ The Sahlgrenska Academy, University of Gothenburg Gothenburg Sweden

## 1 OVERVIEW

As stated by the author in the preface, *The Physics of Radiation Dosimetry*, published by AIP Publishing in 2020, is a result of lecture notes and follows the structure of the lectures given in the dosimetry course in a Master of Science Degree program in Medical Physics. The understanding of the concepts and calculations given in the book thus requires previous studies of physics and mathematics on a physics bachelor level.

## 2 ORGANIZATION

The textbook is approximately 260 pages in length and contains 15 chapters, together with three appendices. All chapters are followed by a collection of problems (with answers) and references. At the end of the book, one appendix is giving an overview of radioactive decay including formulation of the basic decay modes and equations giving the activity in single and series decay of radionuclides. A second appendix lists physical constants and conversion factors, followed by a third appendix giving values of the Klein–Nishina cross sections. An index is included at the end of the book.

## 3 CHAPTER CONTENTS

In order to arrive at the definition of absorbed dose, Meli starts by introducing basic concepts in quantum mechanics and applications in atomic and nuclear physics. This chapter can be understood without prior knowledge in quantum mechanics, and for students at a bachelor level, it provides a good refresher of the concepts needed for the further elaboration of the subject. In Chapter 2, the basic quantities fluence and cross section are introduced. However, fluence is introduced only as a scalar quantity and the alternative representation as a vector quantity is omitted.

Chapters 3–6 deals with various aspects on the interaction of charged particles with matter, eventually leading to calculation of absorbed dose due to these interactions. Generally, the word dose is used for absorbed dose, which is a quantity defined by the International Commission on Radiation Units and Measurements. However, given the topic of the book, the risk for misunderstanding is probably negligible. The possible interaction mechanisms for charged particles are discussed, followed by derivation of the mathematical expression for the cross section for elastic Coulomb scattering. Likewise, inelastic collisions are described, and stopping power (collision and radiative) is defined, as well as the corrections for polarization and energy level for the atomic electron taking part in the interaction (shell correction). Chapter 5 discusses the range for charged particles, including the concept of continuous slowing down approximation range and range scaling. Absorbed dose is defined in Chapter 6 as the average energy absorbed by unit mass. However, the concept of energy imparted, which is a common quantity used in the definition of absorbed dose, is not used in this book. This chapter also includes a discussion of charged particle equilibrium and restricted stopping power.

Having presented the interaction mechanisms and quantities used in the determination of absorbed dose due to charged particle, Meli continues with discussing interaction mechanisms for photons. Starting with a brief introduction to the origin of characteristic X‐rays and Auger electrons, energy relations and cross sections for Compton scattering, Rayleigh scattering, photoelectric absorption, and pair production are derived. Based on the cross sections, the mass attenuation coefficients are derived.

Chapter 9 gives an overview of the production of X‐ray beams, both in the keV and in the MeV range, including the origin of the Bremsstrahlung spectrum. Expressions for absorbed dose in photon beams are given in Chapter 10, derived from the average energy transferred to charged particles in the various interaction mechanisms. The quantity kerma is defined along with a discussion of charged particle equilibrium, both longitudinally and laterally with depth in an exposed medium.

The next topic, discussed in Chapter 11, is how to measure the quantities kerma, exposure, and absorbed dose. Here, the concept of average energy lost by charged particles in creating an ion pair is introduced. The use of ionization chambers is extensively discussed, and examples are given how measurements are performed at U.S. National Institute of Standards and Technology (NIST).

Cavity theories, that is, translating the measured absorbed dose in the detector material to absorbed dose in a medium surrounding the detector, are discussed in Chapter 12. Starting with the theory by Bragg and Gray, the corrections made by Spencer and Attix are presented, as well as the cavity theory proposed by Burlin. The practical use of ionization chambers in terms of effective point of measurement is also covered, followed by examples of dose calculations. An important application of measurements with ionization chambers is in radiation therapy, that, is in photon beams of MeV quality. Calibration factors, along with various corrections, are extensively discussed. There are also other kinds of detectors that can be used, for example, chemical detectors, solid‐state detectors, various films, and thermoluminescent/optically stimulated luminescent materials, and these are covered in Chapter 14.

Interaction of neutrons is discussed in Chapter 15, along with examples of absorbed dose calculations due to neutron interactions in tissue. The technique of measuring absorbed dose from neutrons in the presence of photons by using two detectors is discussed, and an example is given.

## 4 SUMMARY

In my view, this book is a good compilation of the basic topics that need to be covered in a course on external radiation dosimetry. The focus seems to be on using the dosimetry for application in radiation therapy, and for that purpose, the book is a good introduction for a medical physicist. However, the dosimetry of radioactive sources incorporated in the human body is not covered in this book, as, for example, (to some extent) in *Introduction to Radiological Physics and Radiation Dosimetry* by Attix (Wiley‐VCH 2004). Readers who are interested in internal dosimetry will have to find this information elsewhere, for example, in *Fundamentals of Nuclear Medicine Dosimetry* by Stabin (Springer 2008) or *Nuclear Medicine Radiation Dosimetry ‐ Advanced Theoretical Principles* by McParland (Springer 2010). Other textbooks comparable to the one reviewed here are *Applied Physics of External Radiation Exposure* by Antoni and Bourgois (Springer 2017) and *Radiation Physics for Medical Physicists* by Podgoršak (Springer 2016) that also cover, for example, radiation protection (Antoni and Bourgois) and production of radioisotopes (Podgoršak).

